# Mitochondria and Organismal Longevity

**DOI:** 10.2174/138920212803251427

**Published:** 2012-11

**Authors:** Ara B Hwang, Dae-Eun Jeong, Seung-Jae Lee

**Affiliations:** 1 Division of Molecular and Life Science, Pohang University of Science and Technology, Pohang, Kyungbuk, South Korea; 2 School of Interdisciplinary Bioscience and Bioengineering, Pohang University of Science and Technology, Pohang, Kyungbuk, South Korea; 3 World Class University Information Technology Convergence Engineering, Pohang University of Science and Technology, Pohang, Kyungbuk, South Korea

**Keywords:** Mitochondria, Aging, Reactive oxygen species, Dietary restriction, Target of rapamycin (TOR).

## Abstract

Mitochondria are essential for various biological processes including cellular energy production. The oxidative stress theory of aging proposes that mitochondria play key roles in aging by generating reactive oxygen species (ROS), which indiscriminately damage macromolecules and lead to an age-dependent decline in biological function. However, recent studies show that increased levels of ROS or inhibition of mitochondrial function can actually delay aging and increase lifespan. The aim of this review is to summarize recent findings regarding the role of mitochondria in organismal aging processes. We will discuss how mitochondria contribute to evolutionarily conserved longevity pathways, including mild inhibition of respiration, dietary restriction, and target of rapamycin (TOR) signaling.

## I INTRODUCTION

Mitochondria are implicated in many important physiological processes, including metabolism, signaling, apoptosis, cell cycle, and differentiation. In particular, mitochondria are responsible for the production of cellular energy by generating ATP through the electron transport chain (ETC) located on the inner mitochondrial membrane. The ETC system consists of five protein complexes, I to V. Complexes I–IV transfer high-energy electrons and generate a proton gradient across the inner membrane, whereas complex V is an ATP synthase, which generates ATP by harnessing the proton gradient. Because of their importance in cellular physiology, defects in mitochondria are associated with various human diseases [1]. In addition, many studies have shown that mitochondria play a central role in aging.

The free radical theory of aging, which was first proposed by Harman in 1950s, suggests that the main cause of aging is the accumulation of damage resulting from the production of toxic reactive oxygen species (ROS) [2]. This theory has been refined as the mitochondrial theory of aging (or oxidative stress theory of aging), as mitochondria have been shown to be the main source of cellular ROS [3]. According to this theory, as an organism grows older, mitochondria accumulate oxidative damage due to the production of ROS during electron transport for ATP generation. This process in turn causes further mitochondrial dysfunction, as ROS are highly reactive and destroy macromolecules such as proteins, lipids, and DNA. Therefore, with time, the functions of cells and organisms deteriorate, causing aging and eventual death. Consistent with this theory, a growing body of evidence suggests that perturbation of mitochondrial function alters the rate of organismal aging. For example, mice engineered to have high mutation rates in their mitochondrial DNA (mtDNA) display accelerated aging phenotypes [4]. In addition, overexpression of antioxidant enzymes, such as catalases or superoxide dismutases, has been shown to increase the lifespan of *Drosophila* and mice [5, 6].

Despite the popularity of the mitochondrial theory of aging, many recent reports also provide data contradicting this theory. For example, deletion of superoxide dismutase genes does not shorten the lifespan of the nematode *Caenorhabditis elegans* [7-9]. In addition, recent reports suggest that ROS can actually have beneficial effects on longevity [10-12]. Moreover, other studies have shown that a mild reduction in mitochondrial function promotes longevity in model organisms [13-22]. Thus, the relationship between mitochondria and aging appears to be very complicated.

In this review, we will discuss recent findings regarding how mitochondria contribute to the aging process. In particular, we will focus on the roles of mitochondria in evolutionarily conserved signaling pathways that influence lifespan at the organismal level, including dietary restriction (DR), the TOR (target of rapamycin) signaling pathway, and mild inhibition of mitochondrial respiration. 

## II LIFESPAN EXTENSION BY REDUCED MITOCHONDRIAL RESPIRATION

### 1 Extension of Lifespan by Mild Inhibition of Mitochondrial Respiration is Evolutionarily Conserved

Because mitochondria are essential for many biological processes, it is not surprising that severe mitochondrial dysfunction leads to lethality, developmental arrest, or premature aging. However, mild inhibition of mitochondrial respiration extends the lifespan of various species, including yeast, *C. elegans*, *Drosophila*, and mice [13-22]. In yeast, genetic disruption of mitochondrial function by mutations in mtDNA or in genes encoding mitochondrial ETC components in some cases extends replicative lifespan [15], which is defined as the number of daughter cells produced by a given mother cell prior to senescence. In addition to yeast, the lifespan of the roundworm *C. elegans*, a well-established genetic model organism for aging research, has been shown to be increased by a number of mutations in ETC components [13, 14, 16, 23]. The *clk-1* mutant is one of the first discovered long-lived *C. elegans* mutants and has a mutation in an ortholog of COQ9, a mitochondrial hydroxylase that is required for the synthesis of ubiquinone [13, 24]. *clk-1* mutants have defects in mitochondrial respiration due to defects in electron transfer from ETC complex I to complex III, which requires ubiquinone [25, 26]. Subsequently, it was shown that mutations in either *isp-1* (a component of complex III) or *nuo-6* (a mitochondrial NADH dehydrogenase) cause longevity phenotypes [16, 23]. Moreover, lifespan extension by inhibition of the mitochondrial ETC in *C. elegans* has been confirmed in numerous studies including several genome-wide screening experiments using longevity-inducing RNA interference (RNAi) [17, 18, 27] (also see the accompanying review by Bennett *et al.* in this issue [28]). In these studies, mitochondrial components usually comprise the majority of RNAi clones that lengthen lifespan in *C. elegans*. Thus, the lifespan-extending effects of a reduction in mitochondrial ETC components are generalized phenomena in* C. elegans*.

Lifespan extension by inhibiting mitochondrial respiration has been shown to be evolutionarily conserved in both *Drosophila* and mice. RNAi knockdown of several genes encoding components of ETC complexes I, III, IV, or V extends the lifespan of *Drosophila* [21]. In mice, heterozygous knockout of *mClk1* (the mouse ortholog of *clk-1*) extends lifespan in mice with different genetic backgrounds [19]. In addition, knockout of *Surf1* (surfeit locus protein 1), which encodes an inner mitochondrial membrane protein required for the biogenesis of the cytochrome c oxidase (COX) complex, extends the lifespan of mice [20]. Together, these studies demonstrate that longevity caused by inhibition of mitochondrial respiration is conserved across different phyla.

### 2 Metabolic Changes Underlie the Longevity Caused by Mitochondrial Respiration Defects

**Fig. (1):**
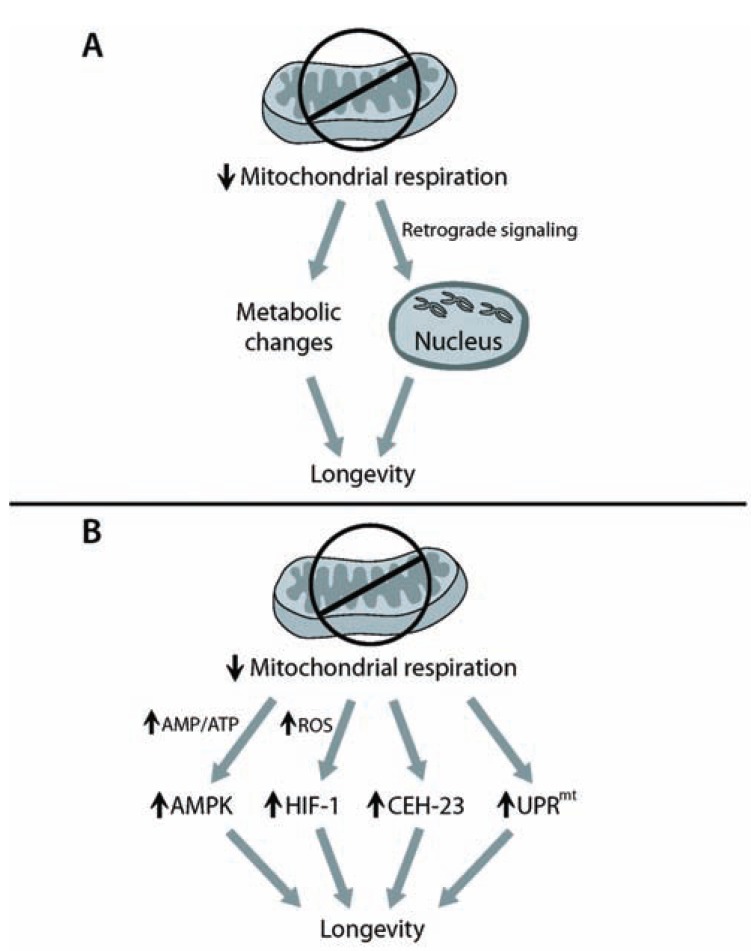
**Lifespan extension by inhibition of mitochondrial respiration.** (**A**) Reduced mitochondrial respiration results in metabolic changes, which contribute to longevity. In addition, impaired mitochondrial respiration elicits retrograde signaling that sends signals from mitochondria to nucleus to extend lifespan. (**B**) Key factors that mediate the retrograde signaling to lead to longevity have been identified in *C. elegans*. Inhibition of mitochondrial respiration increases the AMP:ATP ratio, which activates a lifespan-extending protein AMPK (AMP-activated protein kinase). Reduction in mitochondrial respiration extends lifespan by elevating the level of ROS (reactive oxygen species), which increases the activity of HIF-1 (hypoxia-inducible factor). Induction of CEH-23 (*C. elegans* homeobox 23) and UPR^mt^ (mitochondrial unfolded protein response) mediates the longevity by defects in mitochondrial respiration. How these factors interact with one another is currently unknown.

The “rate of living” theory is one of the first theories of aging proposed by Pearl in the early 20th century [29]. This theory suggests that organisms have a finite number of breaths, and therefore metabolic rate inversely correlates with lifespan. This theory predicts that slow metabolism is linked to slow aging and that this in turn will confer long lifespan. Since mitochondria are essential for energy production and cellular respiration, reduced mitochondrial function is expected to decrease cellular metabolic rates. Hence, one of the simplest hypotheses regarding longevity caused by inhibition of mitochondrial respiration is that such inhibition slows metabolic rate and therefore slows aging. Consistent with this idea, inhibition of mitochondrial respiration not only increases lifespan but also reduces body size, brood size, and behavioral rates of *C. elegans*. Rea *et al*. showed that lifespan, body size, and behaviors are affected coordinately, by performing dose-response knockdown experiments of *C. elegans* ETC components using RNAi dilution methods [30]. These results suggest that physiological processes including aging are delayed by the inhibition of mitochondrial respiration. In addition, ATP levels and oxygen consumption rates were both shown to be reduced by mutations or RNAi knockdown of mitochondrial ETC components [14, 17]. Zuryn *et al*. further showed that a starvation-like global metabolic pathway was altered by RNAi knockdown of mitochondrial ETC components in *C. elegans* [31]. Interestingly, there is a clear negative correlation between lifespan and metabolic rates of different species; small animals have high metabolic rates and short lifespan, and large animals have low metabolic rates and long lifespan [22]. This finding is consistent with the idea that organisms with reduced mitochondrial respiration may have long lifespan because of slower biological processes, including metabolism and aging (Fig. **1A**).

In contrast to this correlation between slow developmental and behavioral phenotypes and longevity caused by inhibition of mitochondrial respiration in *C. elegans*, many studies suggest that longevity caused by reducing mitochondrial respiration does not have to be coupled with other “slow” phenotypes, such as slow development and slow behaviors. For example, inhibiting mitochondrial respiration extends lifespan without influencing the growth or behavior of either flies or mice [19, 21]. In respiration-defective *C. elegans*, a long lifespan phenotype can be suppressed by genetic modulation without affecting other phenotypes [11]. Alternatively, a slow developmental phenotype in *C. elegans* with reduced respiration can be suppressed, while longevity is not affected [16]. In addition, reducing ATP synthesis by RNAi knockdown of mitochondrial ETC components can be uncoupled with longevity. This was first shown by Dillin *et al*. using temporal RNAi knockdown methods in *C. elegans* [17]. These authors showed that RNAi knockdown of the mitochondrial respiration gene *cyc-1* (cytochrome c) during juvenile larval development extends lifespan, whereas reducing mitochondrial respiration during adulthood decreases ATP synthesis without increasing lifespan [17]. Thus, reduced metabolism such as decreased ATP production is insufficient for the longevity of mitochondrial respiration-impaired *C. elegans*. Together, these results are inconsistent with the idea that the “rate of living” is slowed in organisms with reduced mitochondrial respiration via a simple reduction in metabolic rates. It is likely that the mode of regulation of lifespan by mitochondrial respiration defects differs from the modes of regulation of growth, metabolism, and behavior.

### 3 Impaired Mitochondrial Respiration Elicits Retrograde Signaling in Yeast and *C. elegans*

If the longevity conferred by the inhibition of mitochondrial respiration is not directly caused by passive slowing of physiological processes, how is lifespan extension regulated? Several studies suggest that mitochondria send regulatory signals to the nucleus to exert a longevity response (Fig. **1A**). This signaling from mitochondria to the nucleus is referred to as retrograde signaling; signaling from the nucleus to mitochondria has been predominantly studied and referred to historically as anterograde signaling. Jazwinski’s group first showed that retrograde signaling mediates an increased replicative lifespan in mitochondrial respiration mutant yeast [15]. They showed that the activation of retrograde signaling correlates with the increased replicative lifespan of yeast. Moreover, RTG2 (retrograde regulator-2), a critical regulator of retrograde signaling, is shown to be required for this longevity [15]. The role of retrograde signaling in lifespan regulation by mitochondrial respiration appears to be evolutionarily conserved. In *C. elegans*, Cristina *et al*. performed a genome-wide microarray analysis to compare the gene expression patterns of long-lived *clk-1* mutants, *isp-1* mutants, and *cyc-1* RNAi-treated animals with those of wild-type control *C. elegans* [32]. They found that mitochondrial respiration impairment in *C. elegans* changes the expression of global nuclear gene expression [32]. Among the genes that are upregulated in the long-lived respiration *clk-1* mutants, two homologous genes *fstr-1* (faster 1: also known as *gfi-1*) and *fstr-2* (faster 2) are partially required for the long lifespan of *clk-1* mutants. Interestingly, knockdown of *fstr-1* and *fstr-2* accelerates the growth and reversed the induction of several nuclear genes in the *clk-1* mutants [32]. Although the mechanisms are still unknown, these findings suggest that *fstr-1* and *fstr-2* mediate mitochondrial retrograde signaling to decrease rates of behavior and extend the lifespan of *clk-1* mutant *C. elegans*.

### 4 Increased ROS Levels Resulting from Mitochondrial Respiration Defects Promote Longevity

What could be the longevity signal generated from mitochondria in which the ETC is mildly inhibited? One such candidate is ROS because mitochondria are the main source of cellular ROS and because it has been shown that defective mitochondrial respiration may trigger ROS production. Mitochondrial ROS are regarded as unwanted and destructive byproducts of mitochondrial electron transfer. The free radical theory of aging (or the oxidative stress theory of aging) proposes that ROS generated from mitochondria are the main cause of aging [2, 3]. However, many studies have shown that ROS can act as cellular signaling molecules or second messengers [33, 34]. In addition, recent reports suggest that ROS can actually promote organismal longevity. For example, treating *C. elegans* with low doses of paraquat or juglone, chemicals that generate ROS *in vivo*, promotes long lifespan, whereas high doses of these chemicals shorten lifespan [10-12].

Hekimi’s group and our group have shown that increased ROS levels in mitochondrial respiration mutants promote long lifespan [11, 12]. These two studies independently showed that long-lived mitochondrial respiration *C. elegans *mutants indeed contain higher levels of ROS than wild-type animals. Antioxidant treatment abolishes the long lifespan caused by the inhibition of mitochondrial respiration, suggesting a requirement of elevated ROS levels for a long lifespan [12]. ROS appear to act as longevity retrograde signals generated by mitochondria, because ROS increase the activity of hypoxia-inducible factor 1 (HIF-1), a nuclear transcription factor, and this in turn leads to long lifespan ([11]; see below). This mechanism may be evolutionarily conserved in mammals because long-lived *mClk1^+/-^* mice have been shown to display elevated ROS levels [35]. It will be interesting to test whether increased ROS levels can actually result in longevity in mammals.

### 5 Discovery of Key Genes Required for Longevity by Reduced Mitochondrial Respiration

How does the mitochondrial retrograde response mediate longevity caused by inhibition of mitochondrial respiration? Factors that relay signals from mitochondria to the nucleus and nuclear transcription factors that govern the expression of lifespan-regulatory genes are expected to mediate this longevity response. Recent studies using *C. elegans* identified such factors that are crucial for retrograde signals that lead to lifespan extension (Fig. **1B**).

#### AMP-activated protein kinase (AMPK) is partially required for the longevity caused by inhibition of mitochondrial respiration.

AMPK, a crucial cellular energy sensor, is activated by increases in the cellular AMP:ATP ratio. It has been shown that activation of AMPK via genetic or pharmacological intervention extends the lifespan of *C. elegans* and mice [36-39]. Since impaired mitochondrial respiration decreases ATP and therefore increases the AMP:ATP ratio, it is possible that AMPK plays a role in the long lifespan of mitochondrial respiration mutants. Consistent with these findings, Curtis *et al*. showed that the AMP:ATP ratio is increased in *isp-1* and *clk-1* mutants and that AMPK is partially required for the longevity caused by *isp-1* or *clk-1* mutations [40]. Interestingly, AMPK is not required for the slow developmental and behavioral phenotypes of the *isp-1* and *clk-1* mutants. In fact, these “slow” phenotypes are further exacerbated by mutations in the gene encoding the catalytic subunit of AMPK, confirming that the longevity in these respiration mutants does not have to be coupled with other “slow” phenotypes [40]. How AMPK mediates this longevity response is currently unknown. Interestingly, AMPK has been shown to increase mitochondrial biogenesis via the activation of SIRT1 (sirtuin 1; protein deacetylase) and PGC1α/PPARGC1A (peroxisome proliferator-activated receptor gamma coactivator 1 α) in mammals [41]. Therefore, it is possible that defects in mitochondrial respiration activate AMPK as a compensatory response to produce more mitochondria. It will be interesting to determine whether this potential compensatory response contributes to lifespan extension. In addition, because many upstream kinases, such as ribosomal protein S6 kinase and LKB (Lyman Kutcher Burman), and downstream targets of AMPK, such as CREB-regulated transcriptional co-activator (CRTC), have been identified as lifespan regulators [38, 42-45], it will be important to test whether these genetic factors are involved in lifespan extension by inhibition of mitochondrial respiration. 

#### Inhibition of mitochondrial respiration increases HIF-1 activity to extend lifespan.

HIF-1 is a highly conserved transcription factor that acts as a master regulator of cellular adaptation to low oxygen conditions [46]. It has been shown that HIF-1 is crucial for many physiological processes, including angiogenesis, vasculogenesis, axon guidance, pathogen response, and aging [47, 48]. Using a genome-wide RNAi screen to identify new genes that affect HIF-1 activity in *C. elegans*, we found that knockdown of many genes encoding mitochondrial ETC components increases HIF-1 activity [11]. This finding led to subsequent experiments to examine whether HIF-1 activation plays a role in lifespan extension by inhibition of mitochondrial respiration. We found that HIF-1 is required for the long lifespan of *clk-1* and *isp-1* mutants [11]. Moreover, HIF-1 activation is sufficient for long lifespan [11, 49-52], and the longevity caused by HIF-1 activation does not further extend the lifespan of *clk-1* and *isp-1* mutants [11]. Thus, HIF-1 activation is necessary and sufficient for the longevity caused by inhibition of mitochondrial respiration. It was recently shown that HIF-1α is stabilized in long-lived *mClk-1^+/-^* heterozygous knockout mice, suggesting that this regulatory system is conserved in mice [53]. In addition, *mClk1^+/-^* heterozygous knockout mice are protected from cerebral ischemia and reperfusion injury [54]. This finding is intriguing because activated HIF-1 has been shown to play important roles in protecting tissues from ischemia-reperfusion injury [55]. Perhaps mild inhibition of mitochondrial respiration can extend lifespan in mammals by reducing susceptibility to pathological conditions such as ischemia.

#### Homeobox protein CEH-23 mediates the longevity response to impaired mitochondrial respiration.

Walter *et al*. performed an RNAi screen that target transcription factor genes in *C. elegans*, identifying the putative nuclear homeobox protein CEH-23 (*C. elegans* homeobox 23) as a key factor for the increase in lifespan by reduced mitochondrial respiration [56]. They showed that CEH-23 is required for lifespan extension by *isp-1* mutations and is sufficient for the lifespan increase in wild-type animals [56]. The levels of *ceh-23* were increased in *clk-1* and *isp-1* mutants, suggesting that reduction of mitochondrial respiration induces CEH-23 to confer long lifespan. Interestingly, CEH-23 is expressed in restricted tissues, including subsets of neurons and the intestine [56]. Thus, it is possible that retrograde signaling in neurons and/or the intestine is sufficient for the long lifespan of an entire organism, which is consistent with other studies suggesting the involvement of tissue-to-tissue communication for the regulation of longevity by inhibition of mitochondrial respiration [21, 60].

#### Induction of the mitochondrial unfolded protein response (UPR^mt^) mediates the long lifespan caused by inhibition of mitochondrial respiration.

The UPR^mt^ is a stress response that sends signals from mitochondria to the nucleus to induce the expression of mitochondrial protein chaperones [57-59]. Thus, the UPR^mt^ is a good candidate for the retrograde response that mediates longevity caused by impaired respiration. Durieux *et al*. showed that RNAi targeting *cco-1* (cytochrome c oxidase), which extends lifespan, increases the levels of mitochondrial protein chaperones, key effectors for the UPR^mt^ [60]. They further showed that nuclear protein UBL-5 (ubiquitin-like 5), a specific co-factor for the UPR^mt^, is required for the extended lifespan of animals with reduced mitochondrial respiration [60]. Thus, it seems likely that impaired mitochondrial respiration increases the UPR^mt^ to promote long lifespan.

This UPR^mt^ appears to be involved in tissue-to-tissue communication for relaying the longevity signals produced in one tissue to another by the inhibition of mitochondrial respiration. The authors first established that reduction of mitochondrial respiration components in neurons or the intestine is sufficient for increasing the lifespan of entire organisms by performing tissue-specific RNAi experiments through the expression of small hairpin RNA (shRNA) targeting *cco-1* [60]. This result is consistent with studies in *Drosophila* because neuronal knockdown of ETC components is sufficient for lifespan extension in *Drosophila* [21]. Durieux *et al*. further showed that knockdown of mitochondrial respiration in neurons is sufficient for increasing the UPR^mt^ in the intestine, suggesting that the induction of the UPR^mt^ in one tissue sends signals to stimulate the UPR^mt^ in other tissues [60]. These authors subsequently proposed an intriguing model whereby cells experiencing the UPR^mt^ send signals to other tissues to coordinate and delay the aging process of the entire organism. The signals, which they termed “mitokines”, are yet unidentified and will be a focus of future research.

## III DIETARY RESTRICTION AND MITOCHONDRIA

### 1 Dietary Restriction Promotes Longevity

One of the best-conserved interventions extending lifespan across phyla is decreasing total caloric intake without causing malnutrition. In 1935, McCay *et al*. published a paper showing that rats fed a restricted diet live longer than *ad libitum* animals [61]. After this first observation, other organisms, including the yeast *Saccharomyces cerevisiae* [62, 63], *C. elegans* [64, 65], *Drosophila* [66], mice [67], and primates [68] have been shown to live longer with dietary restriction (DR). In general, DR is performed by reducing the composite or overall food sources for these model organisms. DR experiments in yeast are routinely carried out by reducing the concentration of glucose in culture media [62, 63]. Similarly, *Drosophila* DR is achieved by reducing yeast concentration or the total amount of nutrients in media [66]. In *C. elegans*, several methods have been developed to achieve DR, and different genetic factors regulate the DR-induced longevity depending on the specific DR method used. These methods include dilution of the total *E. coli* (food source for *C. elegans *in laboratory) in liquid culture media [64], dilution of the total *E. coli* in solid culture media [69], use of chemically defined axenic media [70], and complete food deprivation after development [71, 72]. Alternatively, genetic mimetics of DR have been well established for *C. elegans* [65]. For example, mutations in the *eat-2* gene, which encodes a subunit of the nicotinic acetylcholine receptor, cause feeding defects; therefore, *eat-2* mutants consume less food than do wild-type animals [73]. To perform DR in mammals such as mice or primates, DR-conditioned groups are provided 60~80% of the total amount of food given to control groups [61, 67, 68]. Generally the mean lifespans of yeast, *C. elegans*, and *Drosophila* form a dome-shape curve, depending on the level of food reduction [62-64, 66, 69]; the intermediary reduction of food usually promotes the longest lifespan, whereas the lowest concentration of food decreases lifespan, likely due to malnutrition.

**Fig. (2):**
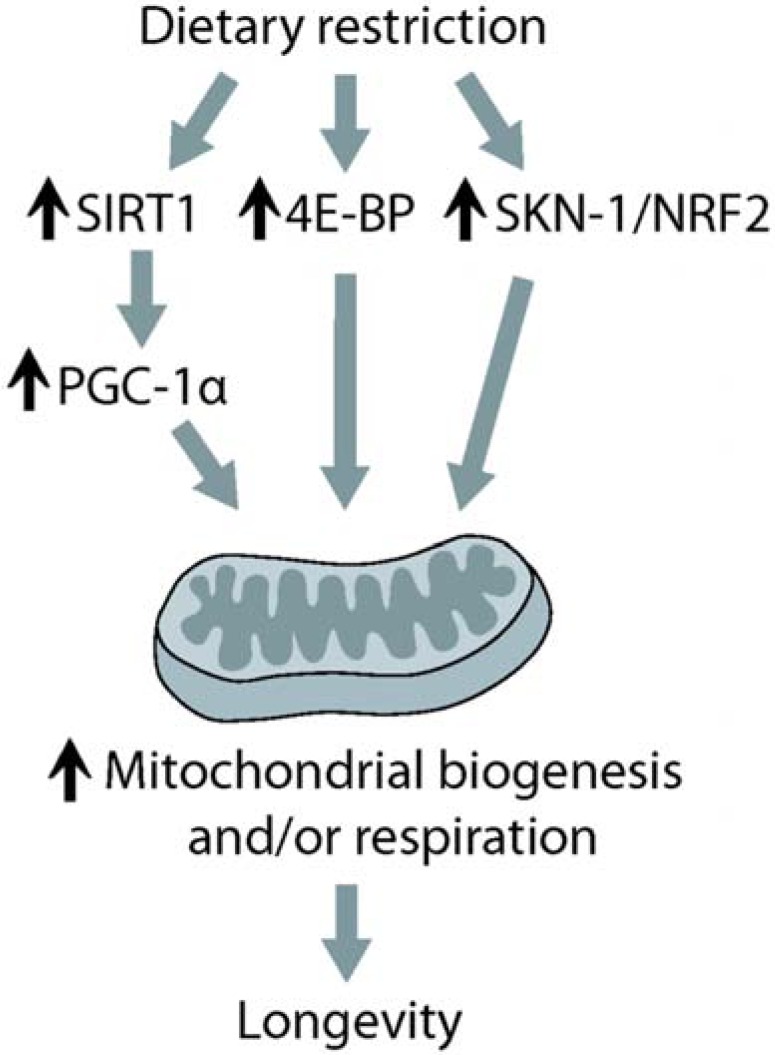
**A model of longevity caused by dietary restriction (DR) via increasing mitochondrial function.** DR increases the activity of several factors crucial for mitochondrial biogenesis and respiration. DR induces SIRT1 (sirtuin 1) activation, which stimulates PGC-1α (peroxisome proliferator-activated receptor gamma coactivator 1 α) activity, to induce genes that are involved in mitochondrial respiration and/or biogenesis. DR increases the activity of 4E-BP (eukaryotic translation initiation factor 4E binding protein), which in turn upregulates the translation of genes encoding respiratory components in *Drosophila*. In *C. elegans*, DR lengthens lifespan through activating SKN-1/NRF2 (nuclear factor-erythroid 2-related factor-2) that enhances mitochondria respiration.

In addition to these DR studies in model organisms, observations in humans with either long- or short-term DR suggest that reducing food levels has similar effects on health and fitness [74, 75]. Therefore, DR has the potential to be used for the improvement of human health. Recent studies have just begun to shed light on the molecular mechanisms by which DR increases lifespan and results in other changes that benefit health. Among these changes, mitochondria appear to play a central role in mediating longevity caused by DR (Fig. **2**).

### 2 DR Enhances Mitochondrial Respiration and Metabolism

One of the observations that has been made repeatedly is that limiting dietary calories increases the rate of mitochondrial respiration. In yeast, *C. elegans* and mice, enhanced mitochondrial respiration as a result of DR has been shown by measuring the increase in total oxygen consumption [70, 76-83]. In addition, these studies showed that DR conditioning does not necessarily diminish ATP production, suggesting that mitochondria may function more effectively to compensate for decreased nutritional uptake by increasing oxygen consumption to generate more ATP [70, 76, 81]. In addition, Lopez-Lluch *et al*. reported that human cells under DR consume less oxygen than do control cells and that total ATP levels are still similar to those of control cells [84]. Thus, mitochondria appear to produce sufficient amounts of ATP even when oxygen consumption rates decrease. These data using a variety of organisms support the idea that reducing dietary calories stimulates mitochondrial respiration to work in more effective ways.

Increased mitochondrial respiration by DR is also shown to correlate with increased expression levels of genes that are involved in mitochondrial respiration. The transcriptional and translational changes in a broad range of genes involved in the mitochondrial ETC by DR have been intensively investigated in yeast. Sharma *et al*. analyzed genome-wide microarray data of dietary-restricted yeast and found that gene ontology terms representing cellular respiration, the mitochondrial ETC, and other mitochondria-related genes were significantly overrepresented [85]. They further confirmed this analysis by performing quantitative reverse transcription (qRT)-PCR for several genes that encode components of the five mitochondrial ETC complexes, and showed that the vast majority of these genes are upregulated more than 2-fold by DR [85]. Another study demonstrated that genes encoding mitochondria-localized proteins including ETC components are significantly induced by DR [86]. In a subsequent study, Choi *et al*. showed that mitochondrial proteins encoded by both mtDNA and nuclear DNA are significantly increased at the post-transcriptional level, as DR leads to an increase in the protein levels of mitochondrial ETC components, whose expression levels are unchanged at the transcriptional level [87]. Therefore, DR appears to increase mitochondrial ETC components at the transcriptional and translational levels, enhancing mitochondrial respiration.

By employing an unbiased approach using yeast, *Drosophila*, and mice, several reports showed that among the genes that are differentially regulated by DR, mitochondrial respiratory pathway genes as well as genes involved in mitochondrial metabolism are over-represented [88-91]. Genome-wide transcriptional profiling data revealed an age-related decline in expression levels of genes involved in mitochondrial biogenesis, mtDNA maintenance, ATP synthase, and mitochondrial protein folding in mouse skeletal muscle [88]. These data are in agreement with previous findings that mitochondrial dysfunction occurs in aged animals [92]. Interestingly, this age-dependent decline in mitochondria-related gene expression is completely or partially prevented by DR begun in early adulthood [88]. In addition, middle age-onset DR prevents the age-related decline in genes engaged in mitochondrial β-oxidation in mouse heart [89]. Similarly, DR induces a broad range of gene expression changes in mouse brain, and COX is among several genes that are induced by DR in the neocortex [93]. In *Drosophila*, DR regulates translational initiation factor 4E-BP (eukaryotic translation initiation factor 4E binding protein) to increase the expression of genes essential for mitochondrial function, including ATP synthesis-coupled electron transport, oxidative phosphorylation, respiratory chain complex I, and mitochondrial ribosome components [91]. After long-term DR, mouse white adipose tissue (WAT) displays increased expression levels of genes involved in overall metabolism, including mitochondrial metabolism [90]. These include genes that are required for the Krebs cycle, fatty acid transport to mitochondria, mitochondrial β-oxidation, the ETC, and oxidative phosphorylation. Moreover, histochemical analyses have shown that COX enzymatic activity is induced by DR in WAT [90]. WAT may play pivotal roles in mammalian lifespan regulation because decreased adipose tissue is associated with increased lifespan in some genetically modified mice [94]. Perhaps overall mitochondrial metabolism and mitochondrial enzymatic activity are enhanced in many tissues including WAT by DR, which may have a crucial role in promoting longevity.

### 3 DR Increases Mitochondrial Biogenesis

How does DR increase mitochondrial respiration? Several reports have demonstrated that mitochondrial biogenesis is enhanced by DR [79, 84, 95, 96]. In mouse WAT as well as in other tissues including brain, liver, heart, and brown adipose tissue, several genes important for mitochondrial biogenesis are upregulated by DR [79]. This was confirmed in human skeletal muscle via qRT-PCR analysis [95], and in a study of an *in vitro* DR model using cultured human HeLa cells [84]. The factors tested for the regulation of mitochondrial biogenesis include PGC-1α/PPARGC1A, Tfam/TFAM (mitochondrial transcription factor A), SIRT1, eNOS (endothelial nitric oxide synthase), and NRF1 (nuclear respiratory factor-1, the master regulator of mitochondrial biogenesis). Among these factors, the role of PGC-1α, which is a critical factor for the induction of mitochondrial biogenesis, has been extensively studied. In addition to the induction of PGC-1α, DR increases the activity and stability of PGC-1α via post-translational modifications by both SIRT1 [97, 98] and GSK3β (glycogen synthase kinase 3 beta) [99]. 

Other molecular markers of mitochondrial biogenesis have been shown to be elevated by DR. Protein levels of core respiratory components such as COX-IV and Cyt c (cytochrome c) are upregulated both in DR-treated mouse tissues and in a DR model using cultured HeLa cells [79, 84]. Relatively short-term DR significantly increases the amount of mtDNA, which reflects the degree of mitochondrial biogenesis, in both mouse tissues (3 months) and muscle tissues of human participants upon DR (6 months) [79, 95]. Finally, electron microscopy analysis showed that the number of mitochondria is increased in liver tissues of DR-treated mice [84]. Thus, DR may augment mitochondrial mass by altering the expression levels of genes or proteins that promote mitochondrial biogenesis. This increase in mitochondrial biogenesis may ultimately assure that the net levels of respiration are maintained and that cells produce sufficient energy under DR. 

### 4 Increased Mitochondrial Respiration is an Underlying Mechanism of Longevity by DR

It has been shown that functional mitochondria are crucial for increases in lifespan by DR. The requirement of mitochondrial respiration for longevity caused by DR was demonstrated by showing that DR-induced longevity in yeast is suppressed by eliminating the ETC component genes *cyt1* (cytochrome c1) or *atp2* (β-subunit of mitochondrial F1-ATPase) [77, 100]. This finding was confirmed by showing that a genetic mimetic of DR in yeast also requires *cyt1* [77]. Lin and colleagues also identified other mitochondrial components that are required for lifespan extension by DR in yeast [101, 102]. They showed that Lat1 (dihydrolipoamide acetyltransferase; a component of the mitochondrial pyruvate dehydrogenase complex), Mdh1 (malate dehydrogenase; a component of the malate-asparate NADH shuttle), Aat1 (aspartate amino transferase; a component of the malate-asparate NADH shuttle), and Gut2 (glycerol-3-phosphate dehydrogenase; a component of the glycerol-3-phosphate shuttle) are required for DR-induced lifespan extension. Moreover, overexpression of these genes is sufficient to promote longevity, which is largely dependent on mitochondrial respiration [101, 102]. Therefore, these findings support the idea that mitochondrial metabolism, including respiration, is crucial for modulating lifespan by DR. In contrast to this suggestion, however, another yeast genetics study proposed that DR may increase lifespan in a mitochondrial respiration-independent manner [103]. These authors used respiration-defective *rho^0^* and single and double *cyt1* mutants in yeast with several different genetic backgrounds, showing that DR increases the lifespan of these respiratory mutants [103]. It is possible that the exact experimental conditions (*i.e.*, the most effective concentrations of glucose needed to perform DR lifespan assays) may vary among laboratories. It is noteworthy that DR induces broad changes to the organism; therefore, not only mitochondrial respiration but other signaling pathways may be crucial for this process.

In *C. elegans*, Bishop and Guarente showed that DR extends lifespan through the activation of the transcription factor SKN-1 (ortholog of human NRF2/nuclear factor-erythroid 2-related factor-2), which increases mitochondrial respiration [80]. The *skn-1* mutation abolishes lifespan extension by reducing bacterial food sources for *C. elegans*, and the neuronal expression of SKN-1 is sufficient to restore the longevity response to DR. Importantly, inhibiting mitochondrial respiration by antimycin treatment, a mitochondrial respiration inhibitor, suppresses the lifespan-extending effects of DR [80]. This is consistent with findings that mitochondria play an essential role in mediating the beneficial effects of DR on yeast longevity.

Schulz *et al*. proposed the functional significance of increased mitochondrial activity to promote longevity by glucose restriction in *C. elegans* [104]. The authors showed that glucose restriction by treating *C. elegans* with 2-deoxyglucose, a glucose analog that inhibits glycolysis, triggers mitochondria to function more efficiently. This in turn produces more ROS from mitochondria. They also demonstrated that increased ROS are required for the lifespan extension because antioxidant treatment suppresses the long lifespan resulting from glucose restriction. The authors proposed that increased ROS from efficiently working mitochondria elicit protective responses that lead to longevity in *C. elegans* [104]. As described above, mild inhibition of mitochondrial respiration increases lifespan via elevated levels of ROS as well [11, 12]. Therefore, these studies provide a potential explanation for how both increased mitochondrial activity by DR and decreased mitochondrial respiration by mutations or RNAi promote long lifespan. In both cases, elevated ROS generated from mitochondria may induce a longevity response. Whether these two phenomena share common downstream effectors is currently unknown and promises to be an active area of future research.

### 5 Resveratrol Mimics DR and Enhances Mitochondrial Function

Although DR has been shown to benefit the health of many species across phyla, decreasing food consumption for humans may not be easy to achieve. As an alternative, many scientists have been trying to identify safe drugs that mimic the effects of DR, and therefore elicit the benefits of DR without dietary changes. Resveratrol (3,5,4’-trihydroxy stilbene) is a small natural compound found in red wine that may increase the activity of the protein deacetylase sirtuins ([105]; see also [106, 107]). Several studies have demonstrated that resveratrol acts as a DR mimetic by increasing mitochondrial activity. Resveratrol treatment improves mitochondrial function in mice fed a high-fat diet [108, 109]. Similar to findings in DR-conditioned mice, transmission electron microscopy analysis showed that liver, muscle, and brown adipose tissue of mice fed a high-calorie diet supplemented with resveratrol display increased numbers of mitochondria compared to the tissues of control animals fed a high-calorie diet without resveratrol supplementation [108, 109]. In addition, Baur *et al*. demonstrated that human cells cultured with serum including resveratrol contain increased numbers of mitochondria [108]. Induction of several transcriptional regulators of mitochondrial biogenesis, including PGC-1α, NRF1, and Tfam, at the transcriptional or translational levels further supports that resveratrol mimics DR in mice by enhancing mitochondrial biogenesis [108, 109]. Microarray analysis showed that resveratrol significantly increases the expression of genes involved in the mitochondrial ETC or oxidative phosphorylation, at least in the muscle tissue of mice fed a high-calorie diet [109]. A recent study using mice demonstrated that SIRT1 is required for the effects of resveratrol on mitochondrial biogenesis [110]. The beneficial effects of resveratrol in mice fed a high-calorie diet were recapitulated in a human trial [111]. Obese but otherwise healthy human participants were given resveratrol supplementation for 30 days and evaluated for changes in their metabolic profiles. Resveratrol supplementation altered expression levels of genes involved in mitochondrial metabolism without resulting in any adverse effects on the health of these participants [111]. The authors found that the levels of phosphorylated AMPK, SIRT1, and PGC-1α, as well as the activity of citrate synthase, were elevated, indicating improvements in mitochondrial function. In addition, the mitochondrial oxygen consumption rate tended to increase after resveratrol supplementation, suggesting increased mitochondrial respiration [111]. Thus, at least in mice fed high-calorie diets and in obese human participants, resveratrol supplementation appears to improve general health, specifically by boosting mitochondrial biogenesis and/or mitochondrial respiration.

## IV TOR SIGNALING AND MITOCHONDRIA

### 1 Inhibition of TOR Signaling Extends Lifespan in Many Species

TOR is a serine/threonine kinase required for cells to sense available nutrients. Sensing nutrient availability is crucial for cells to exert physiological responses to external nutritional conditions such as starvation [112]. TOR phosphorylates many proteins including ribosomal protein S6 kinase and 4E-BP, which play crucial roles in promoting mRNA translation, cellular growth, and division under nutrient-rich conditions. Phosphorylation of S6 kinase by TOR leads to increased mRNA translation via an increase in ribosomal biogenesis [113]. TOR phosphorylates 4E-BP, which leads to dissociation from eIF4E (eukaryotic initiation factor 4E) and in turn results in translation initiation [114, 115]. Several proteins, including regulatory-associated protein of mTOR (raptor) and rapamycin-insensitive companion of mTOR (rictor), physically interact with TOR to determine its functionality. Raptor interacts with TOR to regulate various cellular processes such as mRNA translation and formation of autophagosomes [116-119], whereas rictor interacts with TOR to regulate cellular processes including the actin cytoskeletal organization [120].

For the last decade, it has been shown that inhibition of TOR signaling extends lifespan in several model organisms, including yeast, worms, flies, and mice. Treatment of yeast, *C. elegans*, *Drosophila*, and mice with the TOR inhibitor rapamycin promotes longevity [121-124]. In addition, genetic perturbation of the TOR signaling pathway has been shown to lengthen the lifespan of these model organisms. Deletion of the yeast *tor1* gene increases replicative lifespan as well as chronological lifespan, which is defined as the fraction of viable yeast in the stationary phase [121, 125]. Knockdown of *let-363*, the *C. elegans* homolog of TOR, during the adult stage increases the lifespan of *C. elegans* [126]. In addition, a heterozygous mutation in *daf-15*, the *C. elegans* homolog of raptor, also extends lifespan [127]. In *Drosophila*, reduction of TOR signaling by the overexpression of tuberous sclerosis complex protein 1/2 (TSC1/2) or a dominant-negative form of TOR extends lifespan [128]. 

Down-regulation of S6 kinase also extends lifespan in several model organisms. Deletion of *sch9* and *rsks-1*, which encode the yeast and *C. elegans* homologs of S6 kinase, respectively, extends lifespan [42, 129, 130]. Reducing mRNA translation by genetic inhibition of translation factors or ribosomal protein subunits also extends lifespan [42, 130-133]. In addition, expression of a dominant-negative form of S6 kinase increases lifespan, whereas expression of a constitutively active form of S6 kinase decreases lifespan in *Drosophila* [128]. Recently, it was shown that an S6 kinase 1 deletion results in lifespan extension in female mice [43]. Taken together, these studies demonstrate that longevity caused by reduction in TOR signaling is evolutionarily conserved.

### 2 Extended Lifespan by Inhibition of TOR Signaling Requires Increased Mitochondrial Activity

**Fig. (3):**
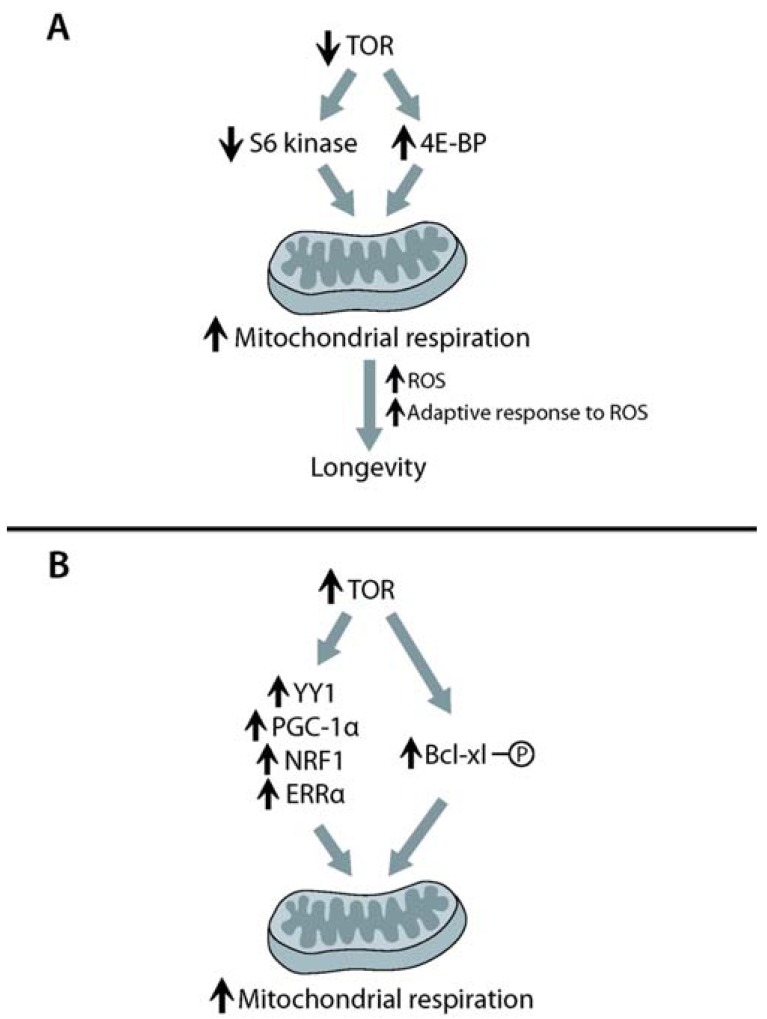
**Two contradicting models of the effects of TOR (target of rapamycin) signaling on mitochondrial function.** (**A**) Reduced TOR signaling increases mitochondrial respiration to induce longevity. Inhibition of TOR signaling downregulates ribosomal protein S6 kinase and upregulates 4E-BP. This leads to overall increase in mitochondrial respiration that may elevate the level of ROS (reactive oxygen species). The production of ROS is likely to confer an adaptive response that is required for longevity. (**B**) TOR signaling positively regulates mitochondrial function. Activated TOR upregulates YY1 (yin-yang 1), PGC-1α, NRF1 (nuclear respiratory factor-1) and ERRα (estrogen-related receptor α), key transcription factors that increase mitochondrial function. YY1 directly binds PGC-1α and TOR to increase the TOR-dependent expression of mitochondrial genes. In addition, TOR phosphorylates Bcl-xl (B-cell lymphoma-extra large), an outer membrane protein of mitochondria, to enhance mitochondrial respiration. It is currently unknown whether these processes shown in (**B**) are involved in lifespan regulation.

Although many components of the TOR signaling pathway have been shown to regulate lifespan, the molecular mechanisms involved in this pathway are incompletely understood. Recent studies suggest that enhanced mitochondrial activity is one of the mechanisms by which reduced TOR signaling results in long lifespan (Fig. **3A**). In yeast, reduced TOR signaling has been shown to increase mitochondrial activity [134]. Long-lived *tor1* deletion mutants or wild-type yeast strains treated with rapamycin display increased oxygen consumption rates, indicating that mitochondrial respiration is enhanced by impaired TOR signaling. Moreover, *tor1* deletion mutations or rapamycin treatment does not extend the chronological lifespan of respiration-defective petite yeast in which mtDNA is mutated [134]. Therefore, reduction of TOR signaling appears to increase mitochondrial activity to extend lifespan. 

Similar findings were made using the long-lived *sch9* (yeast homolog of S6 kinase) deletion mutant yeast. Analysis of genome-wide transcriptional profiles revealed that genes encoding mitochondrial respiratory components are highly enriched among the genes upregulated by the s*ch9 *deletion [135]. In addition, mitochondrial respiration rates of s*ch9 *deletion mutants are increased compared with those of wild-type strains. Increased oxygen consumption and extended chronological lifespan of s*ch9 *deletion mutants are dependent on *hap4* (heme activator protein 4, a component of the transcriptional activator complex, which regulates several genes in the mitochondrial ETC) and *cyt1*. Because *hap4* and *cyt1* are essential for ETC function, these results suggest that increased respiration is required for the extension of chronological lifespan by *sch9 *deletion [135].

How does inhibition of TOR increase mitochondrial activity? Recent studies suggest that inhibition of TOR signaling selectively upregulates the translation of mitochondrial components while reducing general translation [91, 134]. The expression of proteins that are involved in oxidative phosphorylation is increased at the transcriptional and/or translational levels by either *tor1* or *sch9 *deletion mutations [91, 134]. *tor1 *mutation does not change total mitochondrial mass or mtDNA copy number, suggesting that reduced TOR signaling increases the density of the mitochondrial oxidative phosphorylation complex. Pan and Shadel showed that the chronological lifespan-extending effect by *tor1* deletion is abolished in the GS129 yeast strain, which has mutations in the mitochondrial RNA polymerase required for the balanced expression of oxidative phosphorylation components [91, 134]. Thus, increased translation of mitochondrial proteins is required for lifespan extension by *tor1 *deletion mutations. In *Drosophila*, 4E-BP improves mitochondrial function to extend lifespan by DR [91]. A genome-wide translation state array analysis showed that mitochondrial proteins are upregulated at the translational level, whereas general mRNA translation is decreased upon DR [91]. At the molecular level, mitochondrial component-encoding mRNAs that are translationally upregulated upon DR have relatively weak secondary structures in the 5'UTR with short sequences and lower GC content. Overall increases in the translation of mitochondrial components are dependent on 4E-BP, suggesting that the level of mitochondrial proteins is increased by reduction of TOR signaling. Although further research is required to uncover the mechanisms by which reduced translation extends lifespan, a current model suggests that reduced TOR signaling increases the levels of certain mitochondrial proteins, which in turn increase mitochondrial activity to confer lifespan extension.

Impaired TOR signaling increases mitochondrial respiration in mammalian systems as well. S6 kinase 1-knockout mice display enhanced oxygen consumption in general, as well as increases in the number of mitochondria in adipocytes and skeletal muscle compared to those of wild-type animals [136]. In addition, the expression levels of mitochondrial genes that are involved in energy consumption and respiration are increased in adipocytes as well as in the skeletal muscle of S6 kinase 1-knockout mice [136]. Another study showed that adipose-specific knockout of raptor enhances oxygen consumption by altering genes encoding mitochondrial uncoupling proteins (UCPs) in mice [137]. These results indicate that mitochondrial respiration is increased when TOR signaling is reduced in the absence of either S6 kinase 1 or raptor in mammals.

How does increased mitochondrial activity elicit longevity? Recently, Pan *et al*. proposed that increased mitochondrial activity induces an adaptive response to ROS for promoting longevity in yeast [138]. Here, reduction of TOR signaling elevates ROS levels via enhancement of mitochondrial respiration during logarithmic growth. During the stationary phase, however, ROS levels are lower in yeast with reduced TOR signaling than in control yeast, suggesting that increased ROS levels during the logarithmic growth state provide an adaptive response, which is likely a compensatory action that allows yeast to decrease ROS levels during the stationary phase. ROS generation appears to be important for chronological lifespan extension induced by TOR signaling because reduction of ROS by overexpression of the yeast homolog of *sod2* (mitochondrial manganese superoxide dismutase) or by treatment with DNP (dinitrophenol, a chemical uncoupler of oxidative phosphorylation) shortens the chronological lifespan of *tor1* deletion mutants [138]. These findings are consistent with other studies using *C. elegans* showing that a mild increase in ROS is beneficial to long-term survival, whereas high levels of ROS shorten lifespan [10, 11]. 

### 3 Increase in Mitochondrial Activity by TOR Signaling

Contrary to the findings described in the previous section, several recent studies suggested that inhibition of TOR signaling reduces mitochondrial activity (Fig. **3B**). In yeast, analysis of genome-wide transcriptional expression profiles obtained by Wei *et al*. showed that genes involved in mitochondrial respiration are downregulated in *sch9 *and *tor1* deletion mutants [139]. Several studies using cultured mammalian cells and mice also suggest that inhibition of TOR signaling reduces mitochondrial activity. Immortalized Jurkat T cells treated with rapamycin show decreased mitochondrial membrane potential as well as reduced levels of oxygen consumption and oxidative capacity [140]. Furthermore, *TSC2* knockdown, which constitutively activates TOR signaling [141], increases the levels of oxygen consumption and oxidative capacity, whereas knockdown of raptor reduces these levels [140]. These results indicate that enhanced TOR signaling is both necessary and sufficient for increases in mitochondrial respiration. In addition, TOR signaling promotes the expression of *PGC-1α*, *NRF1*, and *ERRα *(estrogen-related receptor α), transcription factors whose downstream targets are mitochondrial genes involved in the control of mitochondrial function [142-145]. Cunningham *et al*. further proposed a mechanism by which TOR signaling increases mitochondrial respiration by identifying YY1**(yin-yang 1) as a key mediator for the interaction between TOR and PGC-1α [145]. The authors showed that YY1 physically binds to mTOR and PGC-1α to increase the mTOR-dependent expression of mitochondrial genes. Rapamycin treatment disrupts the interaction between YY1 and PGC-1α, and therefore reduces the mTOR-dependent expression of mitochondrial genes [145]. Similar to these studies using mammalian cells, skeletal muscle-specific knockout of raptor lowers the expression of* PGC-1α* and its targets in mice [146]. This study also showed that the activity of mitochondrial NADH-dehydrogenase is decreased and that intermyofibrillar mitochondria disappear almost completely in muscles of these knockout mice. Thus, inhibition of TOR signaling in muscle decreases mitochondrial mass as well as mitochondrial activity. Taken together, TOR signaling can enhance mitochondrial functionality via increases in *PGC-1α* both *in vitro* and *in vivo*.

Recent studies also showed a physical association between mitochondria and mTOR [140, 147, 148], suggesting that this interaction is involved in the regulation of mitochondrial activity by mTOR [148]. mTOR is co-immunoprecipitated with Bcl-xl (B-cell lymphoma-extra large) and VDAC1 (voltage-dependent anion-selective channel protein 1) [148], which are mitochondrial outer membrane proteins. This study further showed the significance of the interaction between mTOR and Bcl-xl in mitochondrial function. mTOR directly phosphorylates Bcl-xl [148], whose expression is sufficient to increase mitochondrial respiration [149]. Treatment of ABT-263, a Bcl-xl inhibitor, reduces mitochondrial activity, whereas overexpression of Bcl-xl suppresses a rapamycin-dependent decrease in mitochondrial activity [148]. Thus, mTOR activity influences mitochondrial function through a physical association with mitochondrial proteins, including Bcl-xl.

### 4 Possible Resolutions for Discrepancies Regarding TOR Signaling and Mitochondrial Activity

Thus far, we have described studies showing reductions in mitochondrial function by TOR signaling as well as studies proposing enhancements in mitochondrial function by TOR signaling. How can we reconcile these two possibilities? In yeast, a possible explanation may lie in the differences in growth states. Lavoie and White demonstrated the induction of mitochondrial genes by inhibition of TOR signaling using RNA samples of *sch9*-deletion mutants in early log-phase [135], whereas Wei *et al*. demonstrated the opposite using relatively old *sch9*-deletion mutants to obtain genome-wide transcriptional profiles [139]. This difference in life cycle phase may have resulted in different mitochondrial gene expression and respiration levels, as it was recently shown that reduced TOR signaling affects mitochondrial activity in a growth state-dependent manner [138]. 

In mammals, tissue-specific effects may lead to seemingly contradictory results. Adipose-specific knockout of raptor enhances mitochondrial respiration in adipose tissue, whereas skeletal muscle-specific knockout of raptor reduces overall mitochondrial function in the soleus muscle [137, 146]. Further studies on genetic models of other components in the TOR pathway will determine whether this tissue difference can account for these discrepancies in general.

## V CONCLUSIONS

In this review, we have summarized and discussed findings regarding how mitochondria contribute to the longevity of organisms. Although it is very clear that mitochondria play important roles in aging, the situation appears much more complicated than originally proposed in the mitochondrial theory of aging. Studies using model organisms such as yeast, *C. elegans*, *Drosophila*, and mice have shown that both inhibition and enhancement of mitochondrial function can extend lifespan. Interestingly, recent findings suggest that mild increases in ROS may underlie a common mechanism in these seemingly paradoxical findings. On one hand, mutations in genes encoding ETC components increase ROS. On the other hand, glucose restriction in *C. elegans* or a reduction in TOR signaling in yeast has been shown to enhance mitochondrial efficiency, resulting in an elevation of ROS levels. This increase in ROS appears to mediate retrograde signaling from mitochondria to the nucleus to elicit protective cellular responses, leading to long lifespan. Future research using genetic model organisms will determine the precise mechanisms by which mitochondria act as central organelles that regulate organismal aging.
